# Treatment of chronic HCV infection with DAAs in Rio de Janeiro/Brazil: SVR rates and baseline resistance analyses in NS5A and NS5B genes

**DOI:** 10.1371/journal.pone.0216327

**Published:** 2019-05-07

**Authors:** Vanessa D. Costa, Carlos E. Brandão-Mello, Estevão P. Nunes, Pedro Guilherme Corôa dos Santos Silva, Lia Laura Lewis Ximenez de Souza Rodrigues, Elisabeth Lampe, Francisco Campello do Amaral Mello

**Affiliations:** 1 Laboratório de Hepatites Virais, Instituto Oswaldo Cruz, FIOCRUZ, Manguinhos, Rio de Janeiro, Rio de Janeiro, Brazil; 2 Hospital Universitário Gaffrée & Guinle, UNIRIO, Maracanã, Rio de Janeiro, Rio de Janeiro, Brazil; 3 Instituto Nacional de Infectologia Evandro Chagas, INI/FIOCRUZ, Manguinhos, Rio de Janeiro, Rio de Janeiro, Brazil; Nihon University School of Medicine, JAPAN

## Abstract

The selection of viral strains with resistance-associated substitutions at hepatitis C virus (HCV) NS5A and NS5B genes is considered one of the limiting factors for achieving sustained virologic response (SVR) to combination of direct-acting antivirals daclatasvir (DCV) and sofosbuvir (SOF). Since 2015, this interferon-free regimen has been available in Brazilian clinical routine for treating mono- and HCV/HIV-coinfected patients chronically infected with genotypes 1 and 3. Our aim was to assess SVR rate for Brazilian patients chronically infected with genotypes 1 and 3 after DCV/SOF therapy and the frequency of baseline RASs in HCV NS5A and NS5B genes. Serum samples were collected from 107 monoinfected patients and 25 HCV/HIV co-infected patients before antiviral therapy with DCV/SOF. Genetic diversity of NS5A and NS5B genes was assessed by direct nucleotide sequencing. Overall, SVR rate was 95.4% (126/132), and treatment failure occurred in five monoinfected and one HCV/HIV co-infected patient. NS5A RASs frequency was higher for HCV/HIV patients (28%) than monoinfected patients (16.8%). No difference was evidenced between mono- and HCV/HIV-coinfected groups (15% vs. 16%) regarding NS5B gene. Genotype (GT) 1b strains had significantly more baseline substitutions in NS5A (31.6%) than GT 1a and 3a. At least one primary NS5A RAS described in literature at *loci* 28, 30, 31 or 93 was identified in HCV GTs 1 strains for both groups. As for NS5B, RASs at positions 159 and 316 was observed only in GT 1b strains. This study highlighted that SVR rate in clinical routine in Brazil was similar to randomized clinical trials (89–98%). Our research provided genetic data about the circulation of resistant variants in Brazil. Despite its presence, most of identified baseline mutations did not negatively impact treatment outcome. Genetic diversity of circulating strains suggested that most of the Brazilian HCV chronic carriers are susceptible to new therapeutic regimens including recently approved DAAs.

## Introduction

It is estimated that 71 million people worldwide are chronically infected with hepatitis C virus (HCV) and approximately 2.3 million individuals have HCV/HIV-coinfection [1]. From 2015, epidemiological data demonstrated that about 200,000 hepatitis C cases were notified in Brazil considering anti-HCV or HCV-RNA reactive [2].

The availability of all-oral direct-acting antivirals (DAAs) has further increased sustained virological response (SVR) rates, the primary objective for a successful therapy, usually evaluated 12 weeks after treatment conclusion [3]. These drugs target viral non-structural proteins NS3/4A, NS5A and NS5B. In 2015, Clinical Guidelines for the Treatment of Hepatitis C and Coinfections published by Brazilian Ministry of Health, included the administration of NS5A inhibitor daclatasvir (DCV) in combination with NS5B nucleotide analogue sofosbuvir (SOF) with or without ribavirina (RBV) in a daily regimen. The inclusion criteria for treatment with this DAA regimen was monoinfection with HCV GTs 1, 3 and 4 and an advanced liver fibrosis (treatment-naïve or–experienced) and HIV co-infection in patients infected with HCV GT 1 regardless of liver fibrosis stage [4]. Recently, an update in treatment guidelines has determined the incorporation of new DAAs options for patients infected with GT 1a, such as: (1) NS3/4A protease inhibitor (PI) paritaprevir boosted with ritonavir (PTV/r) plus NS5A inhibitor ombitasvir (OBV) in combination with a non-nucleoside polymerase inhibitor dasabuvir (DSV); (2) SOF plus ledipasvir (LDV) and (3) elbasvir (EBV) and grazoprevir (GZV) [5].

Despite the promising results, the selection of viral strains with resistance-associated substitutions (RASs) at HCV NS5A and NS5B genes can be considered one of the limiting factors for failures to DAA combinations. Viral resistance is characterized by positive selection of viral variants that carry amino acid substitutions responsible to reduce susceptibility to certain drug [6]. Each family of drug exhibits a specific RAS profile that is influenced by HCV GT and is characterized by a difference in the genetic barrier to resistance [7]. Naturally occurring primary RASs in viral subpopulations can affect therapy effectiveness after drug selective pressure [8]. Despite primary RASs usually compromise viral fitness in comparison to wild-type strains, compensatory amino acid substitutions that enhance or restore replication capacity might be selected in resistant variants leading to a viral breakthrough and treatment failure [6].

Considering drug-specific RASs, mutations with high fold change seem to have increased clinical relevance in inducing treatment failure when associated with drug- and host-related factors (presence of cirrhosis and prior HCV treatment) [9]. RASs in HCV NS5A have the major impact on clinical routine and persist for years after treatment [10]. Considering NS5A protein, RASs at amino acid positions M/L28, Q/R30, L31, H/P58, E62 and Y93 can reduce susceptibility to DCV. In relation to the NS5B protein, substitutions at residues L159, S282, V321 and C316 can determine resistance to SOF [7, 9, 11, 12].

Few studies in Brazil have evaluated the effectiveness of DAA therapeutic regimens in Brazilian patients (represented by SVR rate achieved in clinical routine) and the role of RASs in a flaw treatment outcome [13]. Researches in this area can provide background information to Brazilian Ministry of Health on the actual response in practice clinical routine of chronic carriers in the face of financial efforts to provide DAAs regimen as a public health policy. In addition, they provide an evaluation of the circulation of resistant variants in Brazil and its possible impact in therapeutic failure with licensed DAAs and those in phase III clinical trials. The aim of this study was to assess SVR rate after SOF/DCV therapy and the frequency of RASs in HCV NS5A and NS5B genes for mono- and HCV/HIV-coinfected patients chronically infected with GTs 1 and 3a.

## Materials and methods

### Patients

Based on inclusion criteria indicated in 2015 clinical guidelines for using therapeutic regimen with DCV and SOF, this study enrolled 132 NS5A and NS5B inhibitors-naïve individuals chronically infected with HCV GTs 1 or 3a with advanced fibrosis (METAVIR score F3) or cirrhosis (F4), which attended the National Institute of Infectology Evandro Chagas (INI) and Gaffrée & Guinle University Hospital (UNIRIO). Between 2015 and 2017, serum samples were collected from 107 HCV monoinfected patients (1a: 46; 1b: 45; 3a: 16) and 25 from HCV/HIV co-infected patients (1a: 9; 1b: 12; 3a: 4) before antiviral therapy with SOF/DCV with or without RBV (12 or 24 weeks). As determined in 2015 Brazilian Clinical Guideline, RBV addition may be performed especially in patients with cirrhosis, with no response to prior therapy and patients with HCV/HIV co-infection regardless of the degree of fibrosis. Regarding therapy duration, a 12-week regimen was standardized for treatment-naïve GT 1 patients while a 24-week regimen was indicated for GT 1 mono- and HCV/HIV-coinfected patients which had already been experimented with DAAs and/or individuals with cirrhosis Child-Pugh B and C. All GT 3 patients received a 12-week therapeutic regimen. All mono- and HCV/HIV-coinfected patients have completed therapy with SOF/DCV with or without RBV. Fourteen of the monoinfected patients and one HCV/HIV coinfected patient had already been experienced with first-generation PIs boceprevir or telaprevir. Serum samples from six non-responder patients with detectable viral load after the end of therapy period were included in our analyses. SVR rates were accessed 12 weeks after treatment conclusion. HCV viral loads was measured by Abbott Real Time HCV assay (Abbott Laboratories, Chicago, Illinois, USA) with a limit of detection of HCV RNA > 12 IU/mL or > 1.08 Log IU/mL.

### Ethical approval

Samples were collected after obtaining written informed consent from each patient. This study was approved by the ethics committee from Oswaldo Cruz Foundation (CAAE 68116417.2.0000.5248) and by the ethics committee of Gaffrée & Guinle University Hospital (Number 204.445).

### RNA extraction, reverse-transcription and PCR amplification

Viral RNA was extracted from serum samples (200 μL) using High Pure Viral Nucleic Acid Kit (Roche Life Science, Mannheim, Germany) following manufacturer’s recommendations. HCV NS5A (~1600 bp) and NS5B (~1500 bp) genes were amplified by one-step reverse-transcription (RT) with polymerase chain reaction (PCR) followed by a second round of PCR (nested-PCR) using specific primers designed for each subtype (Tables 1 and 2). For first round PCR amplification, reagents from Superscript III One Step RT-PCR system (Thermo Fisher Scientific, Waltham, MA, USA) were used. Second round PCR was accomplished with reagents from Platinum *Taq* DNA Polymerase High Fidelity (Thermo Fisher Scientific).

**Table 1:** Oligonucleotides for NS5A gene amplification of HCV GTs.

Genotype	Technique	NS5A Primers	Sequence (5’–3’)	Genome position	Reference
1a	PCR 1	NS5A_F1	CAGTGCARTGGATGAACCG	6076–6094	[14]
		NS5A_R1	CGAGTTGCTCAGTGCGTT	7671–7688	**--**
	PCR 2	NS5A_F1	CAGTGCARTGGATGAACCG	6076–6094	[14]
		NS5A_R2	TARGACATYGAGCARCACAC	7593–7612	[14]
1b	PCR 1	NS5A_F1	CAGTGCARTGGATGAACCG	6076–6094	[14]
		NS5A_R3	GTCTGTCAAATGTGACTTTCTTCT	7747–7770	[14]
	PCR 2	NS5A_F2	CGGCTGATAGCGTTCG	6093–6108	[14]
		NS5A_R2	TARGACATYGAGCARCACAC	7593–7612	[14]
3a	PCR 1	NS5A_F4	CAGTGGATGAACAGGCTCAT	6097–6116	**--**
		NS5A_R4	CCTCAGCACTACATGGTGT	7663–7681	**--**
	PCR 2	NS5A_F5	GTGGATCAATGAAGACTACCC	6243–6263	**--**
		NS5A_R5	CCTCAGCACTACATGGTGT	7663–7681	**--**

**Table 2:** Oligonucleotides for NS5B gene amplification of HCV GTs.

Genotype	Technique	NS5B Primers	Sequence (5’–3’)	Genome position	Reference
1a	PCR 1	NS5B_F1	CTYAGCGACGGRTCRT	7539–7554	[15]
		NS5B_R1	TCACGGGTRAGGTARTAGAC	8742–8761	[15]
	PCR 2	NS5B_F2	TCGTGTGYTGCTCRATG	7591–7607	[15]
		NS5B_R2	TACCTGGTCATAGCCTCC	8621–8638	[15]
1b	PCR 1	NS5B_F3	TCYTGGTCTACYGTRAG	7551–7567	[15]
		NS5B_R3	AGGARCATGATGTTATCARCTC	8679–8700	[15]
	PCR 2	NS5B_F3	TCYTGGTCTACYGTRAG	7551–7567	[15]
		NS5B_R4	CCTAGTCATAGCCTCCGT	8616–8633	[15]
3a	PCR 1	NS5B_F5	TCTATGTCGTACTCTTGGACCG	7630–7651	--
		NS5B_R5	GGAGTAGGCAAAGCAGCAAAT	9341–9361	--
	PCR 2	NS5B_F5	TCTATGTCGTACTCTTGGACCG	7630–7651	--
		NS5B_R6	CGATCAAGTATCTCCTGGGATTG	8929–8951	--

For HCV NS5A gene, PCR products were obtained with the following conditions: 30′ at 45°C for the reverse transcription followed by 2′ at 94 °C, and then 35 cycles at 94 °C for 15”, 61 °C for 30” and 68 °C for 90”, with an extension at 68 °C for 5′ for GT 1a; for GTs 1b and 3a annealing temperature was 56 °C and 62 °C, respectively. Five microliters of RT-PCR were used in second round PCR with following conditions: initial denaturation at 94 °C for 2′ followed by 30 cycles at 94 °C for 15”, 60 °C for 30” and 68 °C for 2′ for GT 1a; for GTs 1b and 3a annealing temperature was 54 °C and 62 °C, respectively.

For HCV NS5B gene, first and second rounds PCR conditions were the same to HCV NS5A region, except for annealing temperatures which were 53 °C, 54 °C and 62 °C (first PCR) and 53 °C, 53 °C and 62 °C (second PCR) for GTs 1a, 1b and 3a, respectively. PCR products were submitted to 1.5% agarose gel electrophoresis, stained with ethidium bromide and visualized under UV light.

### Nucleotide sequencing

NS5A and NS5B products were purified using High Pure PCR Product Purification Kit (Roche Life Science) and concentration was estimated with Qubit dsDNA BR Assay Kit (Thermo Fisher Scientific) for each sample. Purified products were subjected to nucleotide sequencing reactions in both directions using Big Dye Terminator v3.1 Cycle Sequencing kit (Applied Biosystems, Foster City, CA, USA) according to the manufacturer’s instructions and analyzed on ABI 3730 DNA automated sequencer (Applied Biosystems). After assembly of overlapping contigs, HCV NS5A and NS5B nucleotide sequences were submitted to GenBank database under accession numbers MK135170-MK135301 (NS5A gene) and MK135302-MK135433 (NS5B gene).

### Mutation analyses

Nucleotide sequences were aligned in MEGA version 7.0 [16] together with HCV NS5A and NS5B reference sequences of each HCV GT obtained from the Los Alamos HCV Sequence Database. To evaluate the presence of RASs, it was considered substitutions in amino acid residues described in the literature associated or not with some degree of resistance: M/L28, Q/R30, L31, H/P58, E62 and Y93 for HCV NS5A and L159, S282, V321 and C316 for HCV NS5B protein [7, 9, 11, 12].

### Statistical analyses

Univariate analyses were used to associate the presence of baseline NS5A RASs between HCV GTs. Fisher’s exact test and Pearson chi-square were selected to test the significance level of associations, which was assessed at the 0.05 probability level. Statistical analyses were performed using software Epi Info version 7.1 (Centers for Disease Control and Prevention, Atlanta, GA, USA).

## Results

### Patients characteristics

Clinical, virological and therapeutically baseline data of 132 patients studied are provided in Table 3.

**Table 3:** Therapeutic data for mono- and HCV/HIV-coinfected patients.

Characteristics		Groups			
		Monoinfected (n = 107)	SVR (%)	Coinfected (n = 25)	SVR (%)
Mean age (years) ± SD		63.3 ± 9.9	95.3	56.4 ± 10.4	96
Gender	Female	62 (58%)	96.8	5 (20%)	100
	Male	44 (42%)	93.1	20 (80%)	95
DAA-naïve patients		93 (87%)	95.7%	24 (96%)	95.8%
DAA-experienced patients		14 (13%)	92.9	1 (4%)	100
Genotype	1a	46 (43%)	91.3	9 (36%)	100
	1b	45 (42%)	100	12 (48%)	100
	3a	16 (15%)	93.8	4 (16%)	75
Therapeutic regimen/duration (weeks)	SOF + DCV / 12	24 (22.4%)	91.7	9 (36%)	88.9
	SOF + DCV / 24	1 (0.9%)	100	12 (48%)	100
	SOF + DCV + RBV / 12	62 (58%)	98.4	3 (12%)	100
	SOF + DCV + RBV / 24	20 (18.7%)	90	1 (4%)	100
Mean HCV viral load (IU/mL log10) ± SD		5.7 ± 0.86	95.3	5.9 ± 0.56	96
Hepatic condition	Cirrhotic	90 (84%)	94.4	5 (20%)	80
	Non-cirrhotic	17 (16%)	100	20 (80%)	100

### Post-treatment SVR

Overall, SVR rate in both groups was 95.4% (126/132) after 12 or 24-weeks of treatment. The majority of patients had non-specific symptoms during therapy, such as headache, anemia, nausea and fatigue. Therapeutic failure with SOF/DCV occurred in six (4.6%) individuals, five monoinfected and one HCV/HIV co-infected patient. Clinical and resistance data of non-responders are described in Table 4. Considering monoinfected patients, 102/107 (95.3%) did respond to antiviral therapy. Of these, SVR was achieved by 92.9% (13/14) of the experimented and 95.7% (89/93) of the treatment-naïve patients. For HCV/HIV co-infected patients, SVR rate was 96% (24/25).

**Table 4:** Clinical and resistance data for non-responders patients after DAAs therapy.

Characteristics	Patients					
	1	2	3	4	5	6
Age	56	61	76	57	65	56
Genotype	1a	1a	1a	1a	3a	3a
Group	HCV	HCV	HCV	HCV	HCV	HCV/HIV
Therapeutic regimen	S/D/R	S/D/R	S/D/R	S/R	S/R	S/R
Therapy duration (weeks)	24	24	12	12	12	12
Baseline viral load (Log UI/mL)	5.0	6.8	5.7	4.85	6.86	6.36
Hepatic condition	Cirrhotic	Cirrhotic	Cirrhotic	Cirrhotic	Cirrhotic	Cirrhotic
Baseline NS5A RASs	-	-	-	Q30Y**	-	A30S**
Post-treatment NS5A RASs	-	Y93N	M28T, Q30R, E62D*	Q30Y**	-	A30S**
Baseline NS5B RASs	-	-	-	-	-	-
Post-treatment NS5B RASs	-	-	-	-	-	-

S: sofosbuvir; D: daclatasvir; R: ribavirina

*Secondary RASs

**RASs not demonstrated to be clinically relevant due to low evidence *in vivo*

SVR rates in each HCV GTs for both groups were 92.7% (51/55) for GT 1a, 100% (57/57) for GT 1b and 90% (18/20) for GT 3a. For patients with HCV monoinfection, GT 1b had a higher response rate (100%; 45/45) followed by GT 3a (93.8%; 15/16) and GT 1a (91.3%; 42/46). Regarding co-infected individuals, GT 3a had a response rate of 75% (3/4), while for GTs 1a and 1b SVR was 100%.

### Prevalence of pretreatment NS5A and NS5B RASs

In the present study, NS5A RASs frequency was higher in HCV/HIV patients (28%; 7/25) than in monoinfected patients (16.8%; 18/107). Regarding NS5B RASs, similar frequency was found for both groups (16% vs 15%). Filtering the presence of NS5A RASs by HCV GTs, GT 1b strains (NS5A: 31.6%; 18/57) had significantly more resistance mutations when compared to GTs 1a (NS5A: 7.3%; 4/55) and 3a (NS5A: 15%; 3/20) together (*p* = 0.0040). Similarly, resistance analyses for HCV NS5B gene indicated a frequency of amino acid substitutions in GT 1b strains of 35% (20/57) whereas for GTs 1a and 3a no mutations were identified for both groups.

Considering monoinfected group, GT 1b strains had significantly more substitutions than other GTs (*p* = 0.0076). Similarly, it was observed a statistically significant association for the presence of baseline NS5A RASs between GT 1b and 1a (*p* = 0.0164) and for GTs 1b, 1a and 3a (*p* = 0.0171).

Tables 5 and 6 indicate all identified amino acid substitutions in HCV NS5A and NS5B regions for mono- and HCV/HIV-coinfected patients.

**Table 5:** Baseline amino acids substitutions in HCV NS5A gene.

Genotype	Wild-type amino acid	NS5A RASs*		Total	*p*
		HCV	HCV/HIV		
1a	Q30	30Y (1)	-	4/55 (7.3%)	
	L31	31M (1)	-		
	E62	62D (2)	-		
1b	L31	31M (3)	-	18/57 (31.6%)	0.0040
	L28+R30	28M+30Q (5)	28M+30Q (4)		
	L28+R30+L31	28M+R30Q+31M (1)	-		
	R30+ Y93	30Q+93H (1)	-		
	R30+P58	30H+58S (1)	-		
	P58	-	58S (1)		
	Y93	Y93H (2)	-		
3a	A30	-	30S (1)	3/20 (15%)	
	A30+S62	30V+62T (1)	30S+62T (1)		

*Values in brackets represent the number of single or combined RASs found in the study population

**Table 6:** Baseline amino acids substitutions in HCV NS5B gene.

Genotype	Wild-type amino acid	NS5B RASs*		Total
		HCV	HCV/HIV	
1b	L159	159F (1)	159F (1)	20/57 (35%)
	L159+C316	159F+316N (14)	159F+316N (2)	
	C316	316N (1)	316N (1)	

*Values in brackets represent the number of single or combined RASs found in the study population

## Discussion

In 2015, DAAs SOF and DCV were included in Brazilian clinical guidelines for the treatment of HCV chronic infection. This combined therapy had been the most used by hepatologists in clinical routine, due to its effectiveness to different patient profiles: treatment-naive, non-responders to previous therapies and HCV/HIV co-infected individuals. Until November 2015, treatment options available in Brazil were limited to a protocol with "first-wave" PIs directed to patients infected with HCV GT 1. Those who experienced previous failure did not have other therapeutic alternatives since at that period no other DAAs options were available. In general, the real efficacy of DAAs and SVR assessment rates in non-clinical studies in Brazil could only be accessed since 2016. Consequently, the low number of national scientific studies evaluating and comparing their findings with controlled clinical trials whose SVR rates ranged from 89% to 98% depending on the infecting GT and treatment with previous drugs [17]. In the current study, SVR rate at week-12 post-treatment with SOF/DCV with or without RBV was 95.4% (126/132) which means that therapeutic response for patients from two ambulatories in Rio de Janeiro was similar to results from a previous Brazilian research by Cheinquer *et al*. (2017) [13] and a randomized trial performed by Zhang *et al*. (2016) [18] where SVR values higher than 95% were observed indicating that response rates of Brazilian chronic carriers in practical routine were similar to controlled clinical trials. No statistical analyses were done in our study to compare different groups and genotypes as the samples were biased by the inclusion criteria defined by Brazilian clinical guidelines.

Studies indicated that previous treatment with DAAs for a specific gene may represent a negative factor for a future therapeutic success with a DAA directed to the same target likely due to drug-selective pressure [7, 19]. Here, all patients were treated with NS5A/NS5B inhibitors and those experienced were previously treated exclusively with DAAs directed to NS3 region. Due to this, no impact on SVR rates would be expected between naïve or experienced patients, as reflected in our results considering monoinfected group, where no statistically significant difference was observed (95.7% vs. 92.9%, respectively). As for HCV/HIV coinfected patients, response rate observed in the present study (96%) was close to that reported in ALLY-3 clinical trial where SVR was 97% (133/137) for patients treated for 12 weeks with SOF/DCV [20]. The licensing of new DAAs has improved response rates in HCV/HIV co-infected individuals and SVR values became similar to that observed for monoinfected patients [21]. Here, no noticeable SVR difference was identified between monoinfected and co-infected groups (95.3% vs 96%), showing that introduction of more effective DAAs has considerably increased response rates in co-infected individuals, which were around 50% of SVR in previous therapy with pegylated interferon/RBV [22]. Our results highlighted that HIV co-infection no longer represent a limiting factor for therapy success, with current DAAs regimens being very effective in yielding SVR, the major aim of HCV therapy.

SVR rates observed for each GT was 92.7% (51/55), 100% (57/57) and 90% (18/20) for GTs 1a, 1b and 3a, respectively. Our results are similar to that reported by Sulkowski *et al*. (2014) [17] in a randomized multicenter trial where SVR rates for monoinfected patients (naive or experienced) with GTs 1b or 3 was 100% (35/35) and 89% (16/18), respectively.

In the non-responder GT 1a monoinfected patient who failed a 12-week treatment with SOF/DCV/RBV, the primary (M28T and Q30R) and secondary (E62D) mutations observed could be associated to the treatment failure. M28T mutation confers high-level resistance to DCV (fold change > 100) and OBV (fold change > 1000), while Q30R confers a clinical impact due to the high fold-change to DCV, EBV, LDV and OBV [10]. RAS E62D, although not acting in a primary way, may compensate decreased viral fitness of resistant variants enhancing replication levels that might have influenced negative post-treatment outcome. Importantly, other negative predictive factors related to therapeutic failure, such as decompensated liver cirrhosis and infection with GT 1a strain, may also have contributed with the non-response.

Here, we report the presence of RAS Y93N in a HCV NS5A sequence from a non-responder GT 1a monoinfected patient whose serum sample was isolated after treatment with SOF/DCV/RBV. A study from Wyles DL *et al*. (2017) [10] had reported that this substitution led to a 10000-fold reduced susceptibility to DCV, LDV and OBV, three of the main DAAs currently in use in interferon-free combined therapies. Additionally, therapy duration for this patient was 24 weeks due to unsuccessful previous treatment with interferon/RBV and first-wave PI telaprevir. Baseline sample from this patient indicated that mutation Y93N was not identified in HCV strain before therapy suggesting that the presence of selective pressure imposed by DCV might have influenced the emergence of viral populations with RAS Y93N. In addition to the presence of RASs after treatment, other factors such as high infective viral load (6.8 Log UI/mL), cirrhotic condition and infection with GT 1a might have contributed to a negative therapy outcome.

Sarrazin *et al*. (2016) [23] have identified Q30Y variant in 2.0% of HCV GT 1a sequences analyzed prior to NS5A inhibitor treatment. A similar frequency (2.2%) of this mutation was found in our study. The amino acid substitution from glutamine (Q) to tyrosine (Y) has been rarely described *in vivo* for non-responders patients, therefore, additional studies will be needed to evaluate a possible new resistance profile associated with this mutation.

In regard to NS5A sequence from HCV GT 3a, substitution A30S has previously been reported by Malta F. *et al*. (2017) [11] in a viral sequence of a HCV GT 3a/HIV non-responder coinfected patient (baseline prevalence of 6.7%; 1/15) and in an international case report [24]. In the present study, we found RAS A30S in one GT 3a non-responder patient, however, the inexpressive number of GT 3a patients available in our study (n = 4) prevented us to verify whether this substitution is common in HCV strains circulating in Brazil. The amino acid substitution at position 30 could have been related to the therapeutic failure experienced by the patient, but few *in vivo* reports so far limited the possibility of association of this RAS with non-response. The patient also presented other negative predictive factors for non-response to treatment: (1) viral factors: high infective basal viral load (log 6.36) and HCV GT 3a infection; (2) host factors: male and chronic cirrhotic profile; and (3) therapeutic factor: reduced treatment duration (12 weeks) [19]. An update in Brazilian clinical guidelines published in 2018 increased the duration of treatment for cirrhotic patients infected with HCV GT 3 from 12 to 24 weeks [5].

According to previous reports, baseline RASs frequency in HCV NS5A region determined by conventional and next-generation sequencing techniques presented values between 6% and 16% [25–27]. Here, we observed statistically significant association for the presence of NS5A mutations in GT 1b in contrast to GTs 1a and 3a. These mutations, however, did not impact treatment effectiveness since all patients infected with GT 1b strains achieved SVR. This finding could be explained by differences in genetic barrier to resistance for DCV depending on the infecting HCV GT and/or due to concomitantly administration of SOF with DCV in a combined DAA regimen. A previous clinical report suggested that GT 1b strains might accumulate more resistance mutations than GTs 1a and 3a to overcome DAA action [17]. Here, baseline NS5A RASs was found in 16.8% (18/107) of the monoinfected group, a percentage slightly higher than reported in a study whose prevalence of NS5A mutations was 11.5% (18/156) [11]. Our study has found the presence of RASs in 6.5% (4/46) and 28.9% (13/45) for HCV GTs 1a and 1b, respectively. Our findings differed from the proportion of RASs between GTs 1a and 1b reported by Zeuzem *et al*. (2017) [28] in a study with 5397 samples from North America, Europe, Oceania and Asia where a prevalence of 13% for HCV GT 1a and 17.6% for HCV GT 1b was found. Nonetheless, similar to results from Paolluci *et al*. (2013) [29] (GT 1a: 12.5%; GT 1b: 53.3%), here, the frequency of RASs in GT 1b strains was significantly higher than in GT 1a strains (*p*<0.05). Although its higher frequency in GT 1b, impact of the presence of RASs in treatment outcome is considered more evident in patients infected with GTs 1a and 3a [20, 30]. In our study group, NS5A RASs did not seem to have influenced treatment outcome since all HCV GT 1b monoinfected patients have responded to SOF/DCV therapy.

According to Wyles *et al*. (2017) [10] amino acid substitution L31M confers a clinical impact due to the high fold-change observed *in vitro* for DAAs DCV and LDV (fold change > 100). However, our analyses *in vivo* indicated that baseline presence of RAS L31M in HCV strains of GTs 1a (2.2%) and 1b (6.7%) did not influenced treatment outcome since all five patients achieved SVR. Additionally, low prevalence for L31M variant was also observed by Zeuzem *et al*. (2017) [28] (2.3%) and Paolluci *et al*. (2013) [29] (3.1%). Besides RAS L31M, substitution Y93H has also been highly associated with resistance to DCV [31]. Our findings were similar to previous published data since substitution Y93H was found in 6.7% of GT 1b samples [31, 32]. Likewise RAS L31M, presence of substitution Y93H did not affected treatment efficacy in the three patients infected with this variant strain.

Regarding HCV NS5B RASs for monoinfected patients, the high frequency of combination L159F + C316N (31.1%, 14/45) found in strains of HCV GT 1b was similar to results reported by Noble C.F. *et al*. (2017) [33] and Peres-da-Silva A. *et al*. (2017) [15] whose prevalence was 14.2% (8/56) and 25% (13/52), respectively. The occurrence of these RASs did not appear to have influenced therapeutic response after treatment, since all HCV GT 1b-monoinfected patients has achieved SVR. Substitution at residue 282, mainly identified *in vitro* and highly associated with SOF resistance, was not observed for isolates of HCV GT 1b in our study as well as in findings from Castilho M.C. *et al*. (2011) [34] and Costatino A. *et al*. (2015) [35].

Few Brazilian studies have analyzed NS5A and NS5B genes resistance profile for HCV/HIV co-infected patients. Further studies are needed to better understand the circulation of resistant viral subpopulations in this group. Our analyses indicated that NS5A RASs frequency for referred group was 28% (7/25) while for NS5B RASs was 16% (4/25). HCV GT 1b strains had higher frequency of NS5A (41.7%; 5/12) and NS5B (33.3%; 4/12) RASs than GTs 1a and 3a. According to Plaza Z. *et al*. (2012) [36], none of GT 1a NS5A sequences carried substitutions while for HCV GT 1b, double mutation L31M + Y93H, highly associated with resistance to DCV, were detected in the proportion of 1/15 (6.7%). In contrast to these results, RASs combination L28M + R30Q was identified in a greater proportion in HCV GT 1b isolates. Resistance analyses for HCV NS5B region revealed similarity in RAS C316N frequency reported by Plaza *et al*. (2011) [37] (13.3%), Trevino A. *et al*. (2011) [38] (10%) since 8.3% GT 1b sequences had this mutation in the current study.

In conclusion, this study demonstrated that SVR rate in Brazilian patients chronically infected with HCV treated with DAA regimen DCV/SOF (95.4%) was similar to results from randomized clinical trials. Our research also sought to identify the circulation of resistant variants in Brazil and if emerging RASs could have influenced or not in treatment outcome. Here, amino acid substitutions already described in literature and with limited clinical reports *in vivo* were observed. However, most of these baseline substitutions did not seem to negatively impact treatment outcome, especially for GT 1b since all patients achieved SVR, demonstrating the importance of a combined therapy directed to different viral proteins. Our observations about circulation of resistant variants in Brazil suggested that new DAAs combination, such as OBV/DSV/PTV/r, SOF/LED and EBV/GZV, whose efficacy is not diminished by these substitutions, could be licensed and included in future clinical protocols for Brazilian chronic HCV carriers.
